# Oocyte Competence of Prepubertal Sheep and Goat Oocytes: An Assessment of Large-Scale Chromatin Configuration and Epidermal Growth Factor Receptor Expression in Oocytes and Cumulus Cells

**DOI:** 10.3390/ijms25084474

**Published:** 2024-04-18

**Authors:** Mònica Ferrer-Roda, Dolors Izquierdo, Ana Gil, Maria Emilia Franco Oliveira, Maria-Teresa Paramio

**Affiliations:** 1 Department of Animal and Food Science, Veterinary Faculty, Autonomous University of Barcelona, 08193 Barcelona, Spain; monica.ferrer@uab.cat (M.F.-R.); dolors.izquierdo@uab.cat (D.I.); ana.gil@uab.cat (A.G.); m_emiliafraoli@yahoo.com.br (M.E.F.O.); 2 School of Agricultural and Veterinarian Sciences, São Paulo State University, São Paulo 14884-900, Brazil

**Keywords:** oocyte, goat, sheep, germinal vesicle, EGF receptor

## Abstract

The oocyte competence of prepubertal females is lower compared to that of adults, mainly because they originate from small follicles. In adult females, the germinal vesicle (GV) and epidermal growth factor receptor (EGFR) have been associated with oocyte competence. This study aimed to analyze GV chromatin configuration and EGFR expression in prepubertal goat and sheep oocytes obtained from small (<3 mm) and large (≥3 mm) follicles and compare them with those from adults. GV chromatin was classified from diffuse to condensed as GV1, GVn, and GVc for goats and NSN, SN, and SNE for sheep. EGFR was quantified in cumulus cells (CCs) by Western blotting and in oocytes by immunofluorescence. Oocytes from prepubertal large follicles and adults exhibited highly condensed chromatin in goats (71% and 69% in GVc, respectively) and sheep (59% and 75% in SNE, respectively). In both species, EGFR expression in CCs and oocytes was higher in prepubertal large follicles than in small ones. In adult females, EGFR expression in oocytes was higher than in prepubertal large follicles. In conclusion, GV configuration and EGFR expression in CCs and oocytes were higher in the large than small follicles of prepubertal females.

## 1. Introduction

Juvenile In Vitro Embryo Transfer (JIVET) is a promising technology to increase genetic gain by shortening the generational interval. In sheep, using embryos produced from 4-week-old females reduced the generation interval to 6 months, compared with 12 months using Multiple Ovulation and Embryo Transfer (MOET) [1]. JIVET in Merino sheep breeding programs improves genetic gain by 21% in meat and 33% in wool production [2]. Oocyte competence/quality is the major limiting factor in the development of in vitro-produced embryos. In small ruminants, we concluded that oocytes coming from prepubertal or juvenile females have lower competence for embryo development than those from their adult counterparts. This reduced competence is related to the small follicles from which the oocytes come from, mainly follicles smaller than 3 mm in diameter. An average of 6.05 oocytes per ovary of juvenile goats were selected based on morphology, from which 0.82 were recovered from follicles > 3 mm. In our laboratory, we observed a positive and direct relationship among follicle diameter, oocyte diameter, and embryo developmental competence both in sheep and goats. Further, we concluded that blastocyst development of follicles smaller than 3 mm in prepubertal goats was significantly lower compared to those from adult goats, but these differences disappeared if oocytes were recovered from follicles larger than 3 mm of prepubertal goats (reviewed by Paramio and Izquierdo [3]).

In cattle, it is a common practice to select follicles with a diameter of 3.0 to 5.0 mm for IVEP, but these oocytes present their germinal vesicle (GV) chromatin in various states of preparedness for GV breakdown (GVBD; reviewed by Gilchrist et al. [4]). During mammalian oocyte growth, the GV undergoes various chromatin modifications and large-scale remodeling to regulate gene expression (reviewed by Tan et al. [5]). Chromatin in the GV is initially decondensed and progressively condenses as the oocyte growths, which is vital to endow meiotic and developmental competence of oocytes. In cattle, it has been suggested that a specific GV chromatin configuration could be related to high oocyte competence [6]. In humans, low chromatin condensation shows a negative correlation with meiotic competence, while an intermediate level shows a positive correlation; therefore, the degree of chromatin condensation could be a predictor of spontaneous maturation in stimulated cycles [7].

Acquisition of oocyte competence also depends on the intricate bi-directional communication axis between the oocyte and follicular somatic cells of the cumulus–oocyte complex (COC) (reviewed by Gilchrist et al. [8]). The epidermal growth factor (EGF) signaling network is essential to regulate oocyte maturation and ovulation. The EGF receptor (EGFR) is responsible for inducing cumulus expansion and oocyte meiotic resumption by downregulating the cyclic nucleotides in cumulus cells that suppress meiotic maturation, 3′,5′-cyclic adenosine monophosphate (cAMP) and 3′,5′-cyclic guanine monophosphate (cGMP) [9]. In addition, EGF signaling is a key regulator of oocyte competence acquisition during the antral phase of folliculogenesis. The oocyte is transcriptionally silent following GVBD and relies on stored mRNA to support early embryo development. EGFR mediates communication between cumulus cells and the oocyte in regulating maternal mRNA translation during folliculogenesis (reviewed by Richani & Gilchrist [10]). COCs from small antral follicles have an underdeveloped EGFR signaling system as a means of restricting the ovulatory signal on them [11], and, it develops, at least in part, by the FSH that stimulates follicular growth to the late antral stage [12]. Thus, COCs progressively acquire EGF responsiveness with advancing folliculogenesis [13]. The cumulus cell acquisition of the EGF receptor’s responsiveness represents a major milestone in an oocyte’s developmental progression and acquisition of developmental competence (reviewed by Richani & Gilchrist [10]).

The objective of this study is to characterize two parameters that are well related to oocyte competence, GV chromatin configuration and EGFR expression, and link them to follicle size and puberty status. Oocytes from prepubertal goats and sheep obtained from follicles smaller or larger than 3 mm were analyzed and compared to large follicles from their adult counterparts. We aimed to determine if oocytes from prepubertal anovulatory females have the same conditions as those from fertile adult females.

## 2. Results

### 2.1. Germinal Vesicle Chromatin Configuration

**Figure 1:**
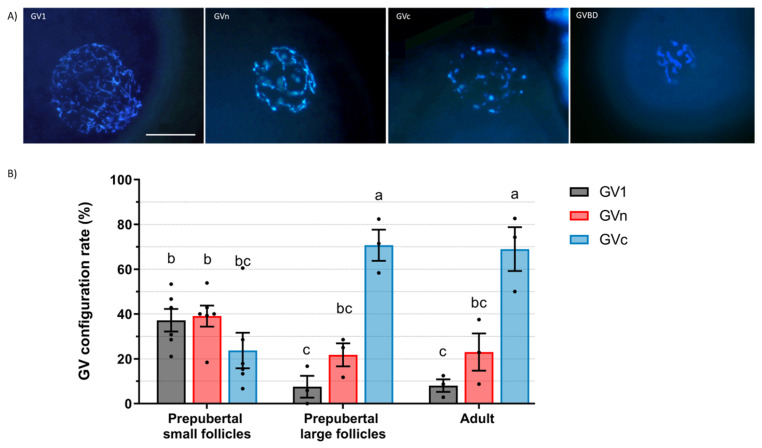
Germinal vesicle (GV) chromatin configuration of caprine oocytes. (**A**) GV configuration and GV breakdown (GVBD) representative images of Hoechst-stained oocytes. Scale bar: 30 µm. (**B**) GV configuration rate (mean ± S.E.M) according to the chromatin configurations from prepubertal small (<3 mm) or large (≥3 m) follicles or from adult follicles (≥3 m). Values with different letters differ significantly (*p* < 0.05). A total of 138, 43, and 66 oocytes for prepubertal small, prepubertal large, and adult follicles were assessed, respectively. Three replicates were carried out.

The GV chromatin configuration at the time of follicular recovery of oocytes from prepubertal small (<3 mm) or large (≥3 m) follicles or from adult follicles (≥3 m) was assessed in goats and sheep. The GV of caprine oocytes presents three chromatin configurations: GV1 has a diffuse chromatin, GVn is an intermediate stage of condensation, and GVc is the most condensed one (Figure 1A). From 165, 59, and 69 stained oocytes (prepubertal small, prepubertal large, and adult follicles, respectively), 86.8%, 72.8%, and 94.6%, respectively, were analyzed as the rest were in GVBD. Prepubertal small follicles did not present statistical differences among oocytes at the GV1 (37.2%), GVn (39.0%) and GVc (23.7%) stages (Figure 1B). Prepubertal large follicles had a greater (*p* < 0.05) proportion of oocytes at the GVc stage (70.7%) compared to GV1 (7.5%) and GVn (21.7%). Adult follicles presented a similar distribution to prepubertal large follicles, with a greater (*p* < 0.05) proportion of oocytes at the GVc stage (68.9%) compared to GV1 (8.0%) and GVn (23.0%). Compared to small follicles, large prepubertal and adult follicles had a higher proportion of GVc oocytes (*p* < 0.05), a similar proportion of GVn, and a lower proportion of GV1 (*p* < 0.05). There were no differences in GV configuration distribution between large prepubertal and adult follicles.

**Figure 2:**
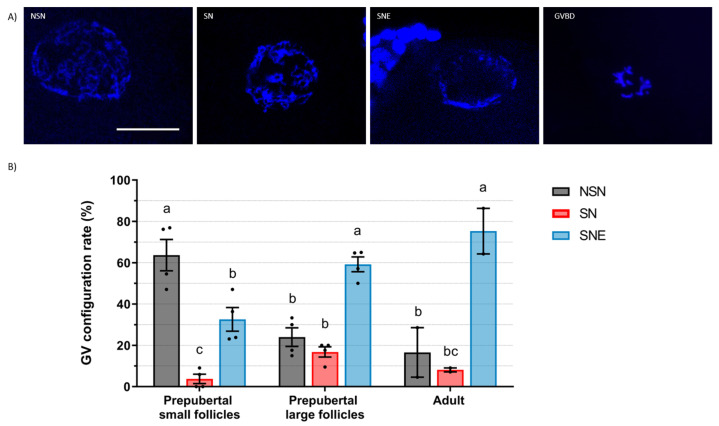
Germinal vesicle (GV) chromatin configuration of ovine oocytes. (**A**) GV configuration and GV breakdown (GVBD) representative images of Hoechst-stained oocytes. Scale bar: 30 µm. (**B**) GV configuration rate (mean ± S.E.M) according to the chromatin configurations from prepubertal small (<3 mm) or large (≥3 m) follicles or from adult follicles (≥3 m). Values with different letters differ significantly (*p* < 0.05). A total of 62, 78, and 36 oocytes for prepubertal small, prepubertal large, and adult follicles were assessed, respectively. Four replicates were carried out for prepubertal groups and two replicates for the adult group.

The GV of ovine oocytes presents three configurations of chromatin condensation: NSN has a diffuse chromatin, SN is an intermediate stage of condensation, and SNE is the most condensed one (Figure 2A). From 66, 78, and 38 stained oocytes (prepubertal small, prepubertal large, and adult follicles, respectively), 93.9%, 100%, and 94.7%, respectively, were analyzed as the rest were in GVBD. Prepubertal small follicles had a greater (*p* < 0.05) proportion of oocytes at the NSN stage (63.7%) compared to the SNE stage (32.6%) and had very few oocytes at the SN stage (3.7%) (Figure 2B). Prepubertal large follicles, on the contrary, had a greater (*p* < 0.05) proportion of oocytes at the SNE stage (59.2%) compared to the NSN (23.9%) and SN (16.8%) stages. Adult follicles presented a similar distribution to prepubertal large follicles, with a greater (*p* < 0.05) proportion of oocytes at the SNE stage (75.3%) than at the NSN (16.6%) and SN (8.1%) stages. Compared to small follicles, large prepubertal and adult follicles had a higher (*p* < 0.05) proportion of oocytes at the SNE stage and a lower (*p* < 0.05) one at the NSN. There were no differences in GV configuration distribution between large prepubertal and adult follicles.

### 2.2. EGFR Expression in Cumulus Cells

**Figure 3:**
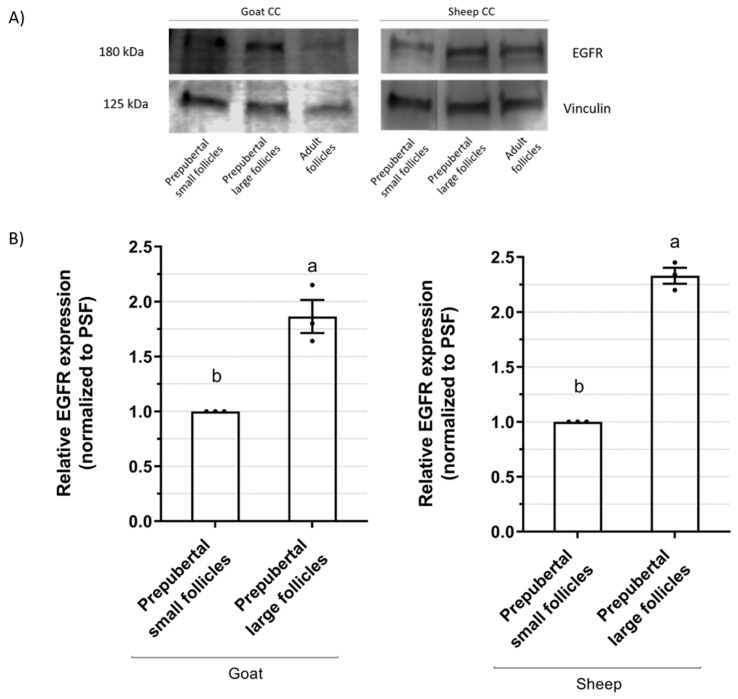
EGFR protein expression in cumulus cells of caprine and ovine COCs from prepubertal small (<3 mm) or large (≥3 m) follicles or from adult follicles (≥3 m). (**A**) Western blot analyses of EGFR expression in goat and sheep cumulus cells. EGFR from 70–80 COCs/replicate was quantified by Western blotting and standardized to vinculin protein levels. Three independent blots were used for relative quantification of prepubertal groups, and a single blot was used for relative quantification of adult groups. (**B**) Relative EGFR expression (mean ± S.E.M) according to follicle diameter of prepubertal females. Values of EGFR expression of each replicate were normalized to the value of their corresponding small prepubertal follicles group (PSF) in the same blot, arbitrarily set at 1. Values with different letters differ significantly (*p* < 0.05).

The EGFR protein expression in cumulus cells at the time of follicular recovery of caprine and ovine COCs from prepubertal small (<3 mm) or large (≥3 m) follicles is presented in Figure 3. Western blot analyses of prepubertal animals showed that the cumulus cells from large follicles had a higher (*p* < 0.05) EGFR expression than the ones from small follicles, both in goats (1.87 ± 0.16-fold difference) and sheep (2.34 ± 0.08-fold difference).

Due to the low number of COCs obtained for adult females, we regarded the EGFR expression of cumulus cells in adult females as a single sample for comparison, although it was not included in the statistical analysis. The sample of cumulus cells from adult follicles showed a 1.82-fold increase in value compared to prepubertal small follicles for goats and a 2.25-fold increase for sheep, which is a value similar to that of prepubertal large follicles for each species.

### 2.3. EGFR Expression in Oocytes

**Figure 4:**
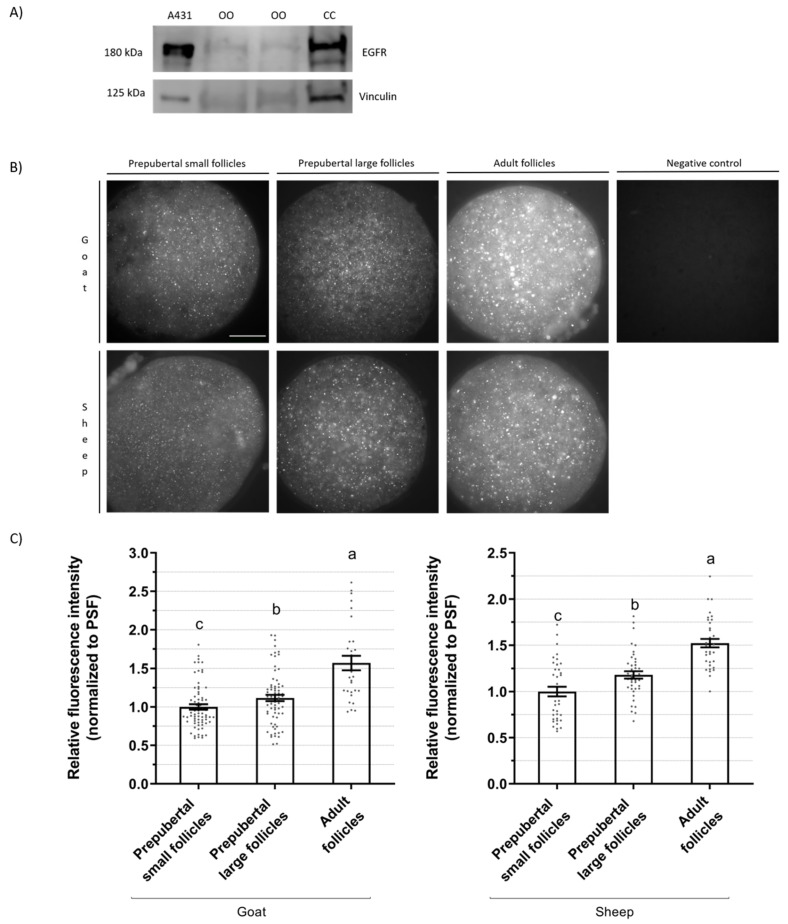
EGFR protein expression in caprine and ovine oocytes from prepubertal small (<3 mm) or large (≥3 m) follicles or from adult follicles (≥3 m). (**A**) Western blot analyses of EGFR expression for A431 cells, a pool of 150 oocytes from prepubertal goats (2 replicates), and the cumulus cells from 70–80 COCs. (**B**) Representative images of immunostained oocytes. Scale bar: 30 µm. Negative controls were prepared omitting primary antibody. (**C**) Relative immunofluorescence intensity (mean ± S.E.M) for EGFR expression in oocytes according to the donor age and follicle diameter. Values for the fluorescence intensity of oocytes stained with EGFR antibody were normalized to the value of the mean of their corresponding prepubertal small follicles group (PSF), arbitrarily set at 1. Values with different letters differ significantly (*p* < 0.05). A total of 69, 71, and 30 oocytes for goat prepubertal small, prepubertal large, and adult follicles were assessed, respectively. A total of 34, 41, and 35 oocytes for sheep prepubertal small, prepubertal large, and adult follicles were assessed, respectively. Three replicates were carried out for goat groups and two replicates for sheep groups.

The presence of the EGFR protein in prepubertal oocytes was verified by Western blotting using a pool of 150 oocytes/replicate in 2 replicates. A431 cell lysate, a human vulval carcinoma cell line that overexpresses EGFR, and cumulus cells were used as a positive control. The immunoblot (Figure 4A) revealed a polypeptide corresponding to EGFR with a molecular mass of approximately 180 kDa for all cell types, as expected [14]. After confirming the protein presence in the oocyte itself, and due to the high number of oocytes needed for the analyses, we established an immunofluorescence imaging protocol to quantify the EGFR abundance in oocytes.

The EGFR protein expression at the time of follicular recovery in caprine and ovine oocytes from prepubertal small (<3 mm) or large (≥3 m) follicles or from adult follicles (≥3 m) is presented in Figure 4. Immunofluorescence analyses of prepubertal oocytes show that the ones from large follicles had a modestly higher (*p* < 0.05) EGFR expression than small follicles, both in goats (1.12-fold) and sheep (1.18-fold). Adult oocytes had an even higher (*p* < 0.05) expression than both prepubertal small and large oocytes (1.57- and 1.52-fold difference, respectively).

## 3. Discussion

We aimed to characterize two parameters related to oocyte competence—GV chromatin configuration and EGFR expression—and to establish their relationship with follicle size and puberty status. Oocytes obtained from prepubertal goats (1–2 months old) and sheep (3–4 months old), originating from follicles smaller or larger than 3 mm, were analyzed and compared to those from large follicles in their adult counterparts.

Our study demonstrates that in prepubertal goats and sheep, oocytes undergo a transition in GV chromatin configuration from diffuse to condensed as the follicle size increases. Goat oocytes display three stages of progressive GV chromatin condensation: GV1, GVn, and GVc. We observed that goat oocytes from prepubertal small follicles were evenly distributed among all GV configurations. In contrast, large follicles, in both prepubertal and adult goats, exhibited highly condensed chromatin (71% and 69% in GVc, respectively). In contrast to our findings, Sui et al. [15] concluded in adult goats that oocytes from follicles larger than 3 mm displayed a higher abundance of the GVn configuration than GVc. These authors categorized GV chromatin based on nucleoli size (GV1-4) and chromatin condensation degree (GV1, GVn, and GVc). They observed that GV1 predominated in follicles up to 0.8 mm in size, GV3c peaked in 2–2.8 mm follicles but declined in follicles >3 mm, and GV3n increased with follicle size and was predominant in follicles >3 mm. Moreover, they discovered that only GV1 and GVn oocytes exhibited transcriptional activity, whereas GVc oocytes did not. They proposed that the GV3c configuration tends towards atresia, while GV3n leads towards ovulation. In our study, perhaps the high proportion of GVc oocytes observed from large follicles were in early atresia, in the case of prepubertal individuals, because they would never ovulate and, in the case of adults, due to the stage of females culled from the herd.

Sheep oocytes display three stages of progressive GV chromatin condensation: NSN, SN, and SNE. We observed that most sheep oocytes from prepubertal small follicles displayed diffuse chromatin (64% in NSN), while most oocytes from large follicles, both in prepubertal and adult sheep, exhibited highly condensed chromatin (59% and 75% in SNE, respectively). Similar to our results, Cocero et al. [16] reported that GV chromatin exhibited similarities in oocytes from adult and 3-month-old sheep when obtained from follicles > 3 mm, with most oocytes in the SNE configuration. Also, Russo et al. [17] described a progressive chromatin condensation process during follicle growth in adult sheep, with SNE oocytes being predominant in 3 to 6 mm follicles.

In bovine, oocytes present four patterns of chromatin configuration, from GV0 to GV3, which exhibit a progressive increase in chromatin compaction [18]. Follicles 0.5–2 mm had mostly GV0 oocytes, but the distribution of GV1-2-3 was equilibrated in follicles of 2–4 mm, 4–6 mm, and >6 mm [19]. After IVF, a higher percentage of GV2 and GV3 oocytes reached the blastocyst stage (22% and 19%, respectively) compared to GV1 oocytes (9%) [18]. In FSH-treated females, Sirard [6] observed that with the coasting of all dominant follicles, the majority of them are at the GV2 stage at the time of collection and, accordingly, their blastocyst rate is close to 75%. If ovaries are recovered at the slaughterhouse, GV2 is found in 30% of oocytes, which is similar to the blastocyst rates found in IVEP programs. In Sirard’s model, the growing follicles would be in GV1, those in the plateau phase would be in GV2, and those in early atresia would be in GV3 with low, high, and medium developmental competence, respectively.

In summary, we observed that, in small ruminants, large follicles exhibit a high proportion of oocytes with highly condensed chromatin in the GVc/SNE configuration. The increase in GV chromatin condensation accompanying follicle growth appears to be independent of reproductive status, as it is similarly observed in both adult and anovulatory prepubertal females.

The second part of this study shows that cumulus cells from goats and sheep acquire progressive EGFR expression as the follicle grows, regardless of reproductive status. In prepubertal females, the EGFR expression in cumulus cells of large follicles is twice that in small follicles, as assessed by Western blot analyses. Similar values of EGFR expression were observed between the large follicles of prepubertal females and the sample of adult females. Few studies have been carried out in small ruminants on this subject. In sheep, EGFR was observed in cumulus cells by immunofluorescence without quantification [20]. In goats, the EGFR was detected in cumulus cells by Western blotting [14], and EGFR gene expression was found to have a positive relationship with oocyte competence [21]. However, the assessment of this protein is of pivotal interest, as it has been demonstrated that signaling through EGFR is necessary for LH to initiate cumulus expansion and oocyte maturation in the follicles of rats, pigs, and humans [22].

Our study shows EGFR expression in the oocytes of goats and sheep. Richani and Gilchrist [10] found that oocytes are not directly responsive to EGF-like peptides, suggesting that they do not notably express EGFR. Additionally, in mice, it has been observed that EGFR is located in mural granulosa and cumulus cells but not in the oocyte (reviewed by Jaffe and Egbert [23]). Our study shows that in prepubertal females, EGFR expression in oocytes from large follicles is higher than those from small follicles, as detected by immunofluorescence analyses. However, the oocytes of prepubertal females obtained from large follicles express significantly lower EGFR than those from adult females. In the present study, EGFR expression in oocytes was further confirmed by Western blotting using a pool of at least 150 denuded oocytes from < 3 mm follicles. In the pig, no expression of EGFR in oocytes was found via Western blotting [24]. In adult sheep, EGFR has been localized by immunochemistry in immature and IVM oocytes, but there is no information about relative protein abundance [20]. In adult goats, protein abundance was higher in meiotically competent oocytes (>3 mm follicles) compared to incompetent ones (<0.5 mm follicles) [14]. According to our results, EGFR is found in ovine and caprine oocytes and cumulus cells, and its expression is positively related to their follicle size and puberty status.

To conclude, oocytes from prepubertal goats and sheep exhibit similar characteristics to those from adult females regarding large-scale chromatin configuration and EGFR expression, which are the two parameters associated with oocyte competence. In prepubertal females, large follicles display a higher percentage of COCs with condensed GVs and a high expression of EGFR in both cumulus cells and the oocytes compared to small follicles. Oocytes from adult females presented a similar GV chromatin stage and a higher EGFR oocyte expression than the oocytes from prepubertal large follicles.

## 4. Materials and Methods

Unless otherwise specified, all chemicals and reagents were purchased from Sigma-Aldrich Corporation (St. Louis, MO, USA).

### 4.1. Oocyte Collection

Goat and sheep ovaries were obtained from a local commercial abattoir. Prepubertal goats were 1 to 2 months old and prepubertal sheep were 3 to 5 months old. Adult goats and sheep were at the end of their productive life, and they were obtained whenever they were available.

Ovaries were retrieved and transported within 2 h to the laboratory at 35 °C in PBS. COCs were recovered according to their follicular diameter. Those from large follicles (≥3 mm) were aspirated with a 20G needle, and the others from small follicles (<3 mm) were collected by slicing previously aspirated ovaries. In adult females, only large follicles (≥3 mm) were aspirated. The collection medium of COCs was HEPES-buffered TCM-199 supplemented with 2.2 mg/mL NaHCO3, 50 ng/mL gentamycin, 11.1 µg/mL heparin; moreover, in order to avoid spontaneous meiotic resumption, 500 µM 3-Isobutyl-1-methylxanthine (IBMX, a phosphodiesterase inhibitor) was added. COCs with a homogenous dark cytoplasm and at least two layers of compact cumulus cells were selected. A selection of just-recovered (immature) oocytes were fixed for further analysis.

### 4.2. Assessment of Oocyte Germinal Vesicle Chromatin Configurations

To evaluate large-scale chromatin configuration, immature oocytes were denuded by pipetting and were fixed in ethanol with 25 µg/mL Hoechst 33,258 overnight. Then, they were covered with Vectashield mounting medium and flattened with a coverslip. GV chromatin was classified based on the degree of condensation. Goat oocytes were classified following Sui et al. [15]’s criteria with a few modifications: GV1 (diffuse filamentous chromatin), GVn (condensed net-like chromatin), GVc (condensed clumped chromatin), and GVBD. Sheep oocytes were classified according to Russo et al. [17] as NSN (diffuse chromatin in the whole nuclear area), SN (condensed chromatin surrounding the nucleolus), SNE (condensed chromatin near the nucleolus and the nuclear envelope), and GVBD.

### 4.3. Epidermal Growth Factor Receptor (EGFR) Quantification

Immature COCs were denuded by pipetting, and EGFR protein was quantified both in oocytes and their respective cumulus cells separately.

#### 4.3.1. Western Blotting

The EGFR from cumulus cells and oocytes was analyzed by Western blotting. After denuding, the cumulus cells from 70 to 80 COCs or a pool of 150 denuded oocytes were recovered, pelleted, and stored at −20 °C until analysis. Cumulus cell and oocyte pellets were disrupted, and protein was extracted in RIPA lysis buffer at 4 °C for 1 h. A431 cell lysate overexpressing EGFR (Servei de Cultius Cel·lulars, Producció d’Anticossos i Citometria, UAB, Bellaterra, Spain) was used as a positive control for the presence of EGFR. Protein extractions were loaded into a 7.5% SDS-PAGE gel (Bio-Rad, Hercules, CA, USA) and transferred to polyvinyl difluoride (PVDF) membranes. For EGFR identification, membranes were blocked in Tris-buffered saline with 0.1% Tween (TBS-T) and 5% BSA for 1 h and incubated overnight at 4 °C with mouse monoclonal anti-EGFR antibody (clone 111.6, MA5-13269, Invitrogen, Waltham, MA, USA) diluted 1:50 in 1% BSA-TBS-T. After three 10 min washes, membranes were incubated with anti-mouse secondary antibody horseradish peroxidase (HRP) conjugated (A16017, Invitrogen) in a 1:2000 dilution. Peroxidase activity was revealed using a WesternSure chemiluminescent substrate kit (Li-Cor, Bad Homburg, Germany) and scanned with C-Digit Blot Scanner and Image Studio Digits v5.2 software (Li-Cor). Band optical density was quantified by Image Studio Digits v5.2 software (Li-Cor). To standardize the results, the same membranes were stripped and incubated with a rabbit polyclonal anti-vinculin antibody (926-42215, Li-Cor, 1:1000), followed by an anti-rabbit HRP secondary antibody (926-80011, Li-Cor, 1:5000). EGFR expression was calculated as the ratio between the optical density of the EGFR band and that of vinculin in the same lane. Values for the EGFR expression (measured in arbitrary units) of each replicate were normalized to the value of their corresponding small prepubertal follicles group in the same blot, arbitrarily set at 1.

Three replicates were conducted for cumulus cells in prepubertal females, whereas due to the low number of COCs obtained for adult females, only one replicate for cumulus cells of adults was performed. Therefore, we regarded the EGFR of CCs in adult females as a single sample for comparison, although it was not included in the statistical analysis.

The band optical density for oocyte samples was too faint to be accurately quantified. Therefore, we developed an immunofluorescence protocol for quantifying EGFR in oocytes.

#### 4.3.2. Immunofluorescence

EGFR in oocytes was quantified by immunofluorescence using a protocol modified from Zhou et al. [20]. Briefly, denuded oocytes were fixed with 4% paraformaldehyde for 15 min. Oocytes were then permeabilized for 30 min in PBS with 0.25% triton, blocked for 1 h in PBS with 10% normal donkey serum, 3% BSA, and 0.15 M glycine, and incubated with mouse monoclonal anti-EGFR primary antibody (clone 111.6, MA5-13269, Invitrogen) in a 1:20 dilution at 4 °C overnight. Oocytes were then washed 3 times for 10 min in PBS with 0.05% Tween and 5% normal donkey serum and incubated for 2 h at 37 °C with secondary anti-mouse antibody Alexa Fluor 488 conjugated (R37114, Invitrogen) in a 1:1000 dilution. DNA was visualized by counterstaining the oocytes with 25 µg/mL Hoechst 33,258 for 10 min. Finally, oocytes were covered with Vectashield mounting medium (VectorLabs, Burlingame, CA, USA) and flattened with a poly-L-lysine treated coverslip, and the slides were stored at −20 °C until analysis the next day. Negative controls were prepared, omitting the primary antibody.

Immunostained oocytes were observed under an epifluorescence Olympus Fluoview 1000 microscope using an excitation wavelength of 488 nm to visualize EGFR and 405 nm to visualize chromatin. Only GV oocytes were photographed for EGFR quantification. A single image per oocyte was taken with a camera Hamamatsu ORCA-Flash4.0 LT Plus, maintaining the same fluorescence parameters and exposure time within the same species. Average fluorescence intensity was measured using ImageJ v1.51h software (National Institute of Health, Bethesda, MD, USA). The ooplasm area was selected, and mean intensity (total intensity/area selected) was quantified. Values for the fluorescence intensities (measured in arbitrary units) of oocytes were normalized to the value of the mean of their corresponding small prepubertal follicles group, arbitrarily set at 1.

### 4.4. Statistical Analyses

Data were analyzed with two-way ANOVA followed by Tukey’s multiple comparison test using SAS 9.4 software (SAS Inst. Inc., Cary, NC, USA). Group was set as the fixed factor and replicate was set as the random variable. Percentage data and data which did not present a normal distribution were square root arcsine-transformed prior to ANOVA. Results were considered statistically significant when *p* < 0.05.

## Data Availability

Dataset available upon request from the authors.
